# Antigenic characterization of influenza viruses produced using synthetic DNA and novel backbones

**DOI:** 10.1016/j.vaccine.2016.05.031

**Published:** 2016-07-12

**Authors:** Pirada Suphaphiphat, Lynne Whittaker, Ivna De Souza, Rodney S. Daniels, Philip R. Dormitzer, John W. McCauley, Ethan C. Settembre

**Affiliations:** a Seqirus (previously Novartis Influenza Vaccines), 45 Sidney Street, Cambridge, MA 02139, USA; b Crick Worldwide Influenza Centre, The Francis Crick Institute, Mill Hill Laboratory, The Ridgeway, Mill Hill, London NW7 1AA, UK

**Keywords:** Synthetic influenza virus, Antigenicity, Hemagglutination inhibition

## Abstract

The global system for manufacturing seasonal influenza vaccines has been developed to respond to the natural evolution of influenza viruses, but the problem of antigenic mismatch continues to be a challenge in certain years. In some years, mismatches arise naturally due to the antigenic drift of circulating viruses after vaccine strain selection has already been made. In other years, antigenic differences between the vaccine virus and circulating viruses are introduced as part of the current system, which relies on the use of egg-adapted isolates as a starting material for candidate vaccine viruses (CVVs). Improving the current process for making vaccine viruses can provide great value. We have previously established a synthetic approach for rapidly generating influenza viruses in a vaccine-approved Madin Darby canine kidney (MDCK) cell line using novel, high-growth backbones that increase virus rescue efficiency and antigen yield. This technology also has the potential to produce viruses that maintain antigenic similarity to the intended reference viruses, depending on the hemagglutinin (HA) and neuraminidase (NA) sequences used for gene synthesis. To demonstrate this utility, we generated a panel of synthetic viruses using HA and NA sequences from recent isolates and showed by hemagglutination inhibition (HI) tests that all synthetic viruses were antigenically-like their conventional egg- or cell-propagated reference strains and there was no impact of the novel backbones on antigenicity. This synthetic approach can be used for the efficient production of CVVs that may be more representative of circulating viruses and may be used for both egg- and cell-based vaccine manufacturing platforms. When combined with mammalian cell culture technology for antigen production, synthetic viruses generated using HA and NA sequences from a non-egg-adapted prototype can help to reduce the potential impact of antigenic differences between vaccine virus and circulating viruses on vaccine effectiveness.

## Highlights

• Synthetic influenza viruses are antigenically similar to conventional reference viruses.
• Use of novel backbones does not affect antigenic characterization.
• Synthetic technology can produce virus candidates for vaccine manufacture that are more representative of circulating strains.

## 1 Introduction

Influenza viruses have a high mutation rate in their RNA genomes and exist as complex quasi-species [1], [2], a property that facilitates their natural drift and continuously challenges vaccine production. Influenza strains that circulate in humans frequently acquire antigenically important mutations to escape immunological pressure, giving rise to new variants that can become dominant and cause seasonal re-infections [2], [3]. These antigenic changes dictate that the influenza vaccine be reviewed bi-annually and updated almost as often. Vaccination is the most effective strategy to protect against seasonal influenza; however, vaccine performance varies from year to year, with decreased effectiveness potentially associated with differences between the antigens in the vaccine and those of circulating strains [4], [5], [6].

Currently, mammalian cells, particularly MDCK cells, are a preferred substrate for influenza virus isolation for surveillance activities due to their high sensitivity to infection [7]. However, only influenza viruses that can be re-isolated and propagated exclusively in embryonated hen's eggs are recommended as CVVs for both mammalian cell-based and egg-based vaccine manufacturing platforms. This standard practice perpetuates the likelihood of producing a vaccine that differs antigenically from circulating viruses.

The variability of virus isolation rates in eggs, particularly for recent H3N2 strains [8], can limit the number of suitable CVVs available in some years. This limitation could lead to a problematic situation as in 2004, when no well-matched H3N2 strain could be isolated in eggs in time to produce a seasonal vaccine, resulting in substantial antigenic difference between the vaccine and the H3N2 viruses in circulation at that time and an associated reduction in vaccine effectiveness [5], [9]. Furthermore, human-derived influenza viruses propagated in eggs undergo selective pressure and can acquire HA mutations that alter their binding specificity from the α-2,6-linked sialic acids that predominate in the upper human respiratory epithelium [10] to the α-2,3-linked sialic acids that predominate in the egg allantoic cavity [11], [12]. Although not all egg adaptation changes in the HA molecule translate to a change in antigenicity, in recent years, the recommended H3N2 and B-Victoria lineage CVVs have exhibited significantly reduced antigenic similarity to circulating strains due to a few egg-adaptive mutations [4], [13], [14].

Although evolutionary drift in circulating viruses cannot be controlled, changes in the antigenic properties introduced as part of current egg-based influenza vaccine production systems can be eliminated. It is known that influenza viruses propagated in mammalian cells often remain genetically and antigenically similar to the virus present in clinical material [15], [16], [17]. Thus, the use of viruses isolated in mammalian cell lines qualified for vaccine production can help maintain antigenic similarity of vaccine strains to circulating viruses [17].

We have developed an efficient synthetic approach for generating high-yielding influenza viruses exclusively in vaccine-qualified MDCK cells [18]. These viruses are produced by reverse genetics from synthetically-derived nucleic acids based on reported HA and NA sequences and combined with optimized backbone gene segments [18]. These high-growth backbones can increase virus rescue efficiency and HA yields [18], and are not expected to impact antigenicity. To demonstrate the ability of this synthetic approach to provide viruses that maintain genetic and antigenic similarity to the intended reference strains, we have performed a study in which the antigenicity of a panel of synthetic viruses covering seasonal A/H1N1, A/H3N2, and B strains was compared to their respective egg- or mammalian cell-grown reference counterparts. The H3N2 subtype was prioritized in this study because H3N2 CVVs have failed to correspond antigenically to circulating strains for the past several years and continue to present a challenge.

## 2 Materials and methods

### 2.1 Cells and viruses

MDCK 33016PF cells were maintained as previously described [18]. Wild-type influenza viruses were isolated from clinical samples by World Health Organization (WHO) National Influenza Centers. Egg-based reassortant viruses were generated at New York Medical College, USA, the National Institute for Biological Standards and Control (NIBSC), UK, or CSL, Australia. All viruses were from stocks held at the Crick Institute, Mill Hill laboratory, UK.

### 2.2 Synthetic DNA

HA and NA segments were assembled as previously described [18], or with the following modifications. Overlapping oligonucleotides were assembled using primers BMP_13 and BMP_14 [18]. PCR products were denatured and re-annealed to form mismatched duplex DNA, followed by incubation with Surveyor nuclease (Transgenomic, Inc.) and Exonuclease III (NEB). Error-corrected DNA was amplified using nested primers BMP_27 (TTGGGTAACGCCAGGGTTTTCC) and BMP_34 (TTCACACAGGAAACAGCTATGACCATGATTA), and purified by ethanol precipitation. Final products were linear gene segments flanked by upstream and downstream regulatory control elements.

### 2.3 Reverse genetics

Synthetic viruses were generated as previously described [18]. Briefly, synthetic HA and NA gene cassettes and plasmids carrying the six backbone genes (PB2, PB1, PA, NP, M, NS) and the plasmid TMPRSS2 (encoding a serine protease [19]) were co-transfected into MDCK cells. Clarified culture medium was harvested at least 72 h post-transfection, and viruses titered by a focus-formation assay [18]. Viruses were passaged up to 3 times in MDCK cells.

### 2.4 Virus sequencing

Viral RNA was extracted using the QIAamp Viral RNA Mini Kit (Qiagen), and cDNA generated using Monsterscript reverse transcriptase (Epicentre). HA and NA genes were amplified using Platinum PCR SuperMix High Fidelity DNA polymerase (Life Technologies), and sequences analyzed by Sanger DNA sequencing (Genewiz, Cambridge, MA).

### 2.5 HI assays

Hemagglutination and HI assays were performed according to standard WHO methods [20]. Four HA units (HAU) of H3N2 strains were tested using 1% suspensions of guinea pig red blood cells and 20 nM oseltamivir carboxylate. HA titers were determined in the presence of the drug. Eight HAU of H1N1 and four HAU of B viruses were tested using 0.75% suspensions of turkey red blood cells without oseltamivir. HI titers were reciprocals of the highest dilutions of sera that inhibited hemagglutination. Post-infection ferret antisera against various reference viruses were treated with receptor-destroying enzyme from *Vibrio cholera* (Cosmos Biomedical, UK).

### 2.6 Ferret inoculation

Post-infection antisera were produced in ferrets (*Mustela putorius furo*) following intranasal instillation of diluted virus under light sedation, and sera were collected under terminal anaesthesia at the Crick Institute Mill Hill laboratory under UK Home Office project license PPL/80/2541 or were made by NIBSC, UK, under UK Home Office project license PIL/80/2530. Other antisera were from the WHO CC at the Centers for Disease Prevention and Control, Atlanta, GA, St Jude's Children's Research Hospital, Memphis, TN, and the Peter Doherty Institute for Infection & Immunity, Melbourne, Australia.

## 3 Results

### 3.1 Generation of synthetic viruses

Synthetic and reverse genetic technologies enable the selection of genomes to generate a new vaccine virus, based on known virus sequences. Three optimized backbones (PR8x, #19, and #21) derived from low pathogenicity strains [18] were used to make influenza A viruses. The PR8x backbone contains six internal genome segments from an MDCK-adapted A/Puerto Rico/8/1934 (H1N1) strain. The #19 backbone contains PB2, PB1, and NP from an MDCK-adapted A/Hessen/105/2007 (H1N1) strain and the remaining segments from PR8x. The #21 backbone contains an A/California/07/2009 (H1N1) PB1 and the remaining segments from PR8x. Influenza B viruses were made using all six backbone segments from B/Brisbane/60/2008. Rescued viruses were passaged up to three times in MDCK cells exclusively. Viruses generated for antigenicity testing covered seasonal influenza strains (A/H1N1, A/H3N2, and B-Victoria lineage), including four egg- and mammalian cell-derived antigen pairs (Table S1). The HA and NA genes of all viruses were confirmed to have 100% genetic identity to the coding sequences used for synthesis (Table S2).

### 3.2 Antigenic characterization of synthetic viruses

We first demonstrated that this MDCK cell-based technology could generate viruses that were antigenically similar to conventional egg-adapted CVVs, even though the synthetic viruses were never passaged in eggs. Synthetic viruses were made using HA and NA sequences from five egg-adapted, high-growth, reassortant CVVs (NIB-74, X-187, IVR-165, X-175, or IVR-164), and virus antigenicity was tested by a one-way HI assay using ferret antisera raised against the egg-adapted CVVs or the corresponding egg-adapted wild-type isolates (Table 1). For all five strains tested, antisera raised against the CVVs recognized the corresponding synthetic viruses at titers ≤2-fold different from the homologous virus titers, regardless of the backbone used. All the synthetic viruses also reacted similarly in HI assays with ferret antiserum raised against the egg-adapted wild-type strains, with titers ≤4-fold different from the homologous virus titers.

Because we aim to generate CVVs that are antigenically identical to any newly isolated wild-type strain by direct rescue on high-growth backbones, we extended our analysis to use HA and NA sequences from H3N2 wild-type isolates, rather than high-growth reassortants. Synthetic viruses were made using HA and NA sequences from two mammalian cell-grown, wild-type isolates, A/Victoria/210/2009 and A/South Australia/3/2011, and from two egg-adapted strains, A/Texas/50/2012 and A/Berlin/93/2011 (Table 2). After one passage in MDCK cells, antigenic characterization was performed by HI tests using ferret antisera raised against the cell- or egg-propagated wild-type strains. Viruses made with the different backbones reacted similarly to a given antiserum, and HI titers obtained from all the tested viruses were within 4-fold of the homologous virus titer.

### 3.3 Synthetic viruses can improve antigenic match to strains that cause human disease

Synthetic viruses can be made using HA and NA sequences from either egg- or mammalian cell-grown isolates, but the ability to genetically match a mammalian cell-grown virus would be expected to improve antigenic similarity to strains circulating in the human population. It has been documented that egg-selected changes in HA for the recent H3N2 and B-Victoria lineage CVVs used in seasonal vaccines have been associated with reduced vaccine effectiveness [4], [13]. Therefore, we used synthetic technology to assess differences in antigenicity between mammalian cell- and egg-derived paired antigens for three recent H3N2 strains and one B-Victoria lineage strain. In the northern hemisphere, A/Victoria/210/2009 was the H3N2 component for the 2010–2011 and 2011–2012 vaccines; A/Victoria/361/2011-like was the H3N2 component for the 2012–2013 and 2013–2014 vaccines; and A/Switzerland/9715293/2013 was the H3N2 recommendation for the 2015–2016 vaccine. B/Brisbane/60/2008 was the B strain component for the 2009–2010, 2010–2011, and 2011–2012 trivalent vaccines, and has been the B-Victoria component for quadrivalent vaccines since 2012–2013. The HA sequences of all mammalian cell- and egg-derived antigen pairs differed by 1-3 amino acids (Table S3). Notably, all the egg-derived H3 antigens contained a G186V mutation that has been shown to improve virus growth in eggs but alter antigenicity relative to an MDCK cell-propagated virus [21]. The egg-derived B/Brisbane/60/2008 HA loses a potential glycosylation site near the receptor binding site. Loss of this glycan upon egg adaptation has also been shown to affect antigenicity [13]. Prior to antigenic characterization, all viruses were assessed for relative growth in MDCK cells to confirm that they could be propagated and potentially used as CVVs (Fig. S1), although infection conditions were not optimized in the study to ensure maximum virus yields.

Synthetic viruses made from either mammalian cell- or egg-derived HA and NA sequences were compared by HI assay to their synthetic counterpart and to conventional reference strains using antisera raised against mammalian cell- and egg-grown reference viruses. As shown in Table 3, Table 4, in most instances we observed differences in reactivity between the cell- and egg-derived antigens when analyzed with ferret antisera raised against the egg-grown viruses, but not when analyzed with antisera raised against the mammalian cell-grown viruses. In particular, ferret antisera raised against the egg-grown vaccine viruses generally recognized synthetic test viruses containing the egg-adapted HA sequence at a titer within 2-fold of the homologous titers, but reacted less well in HI assays (>4-fold decrease) to viruses expressing the corresponding mammalian cell-derived antigens. This experimental design models the current human immunization situation, in which an immune response against an egg-derived vaccine antigen is intended to protect against circulating strains that lack egg adaptations. However, ferret antisera raised against the cell-grown isolates recognized viruses expressing either the cell- or egg-derived antigens. Most viruses reacted to a given antiserum raised against mammalian cell grown viruses within ≤4-fold of the corresponding homologous titer, with the exception of the antiserum that was raised against the cell-grown A/Victoria/361/2011, which produced a low homologous titer of 320 but reacted much better (>4-fold increase) to viruses expressing the egg-adapted antigens (Table 3B). This surprising finding might be attributed to differences in receptor avidity between egg-adapted and cell-grown viruses, which could affect the dynamics of the HI assay. Since recent cell-grown H3N2 viruses appear to have a lower avidity for the sialic acid receptor, more cell-grown A/Victoria/361/2011 virus might have been used in the HI assay to obtain 4 HA units compared to the egg-adapted viruses, therefore requiring more antibody to inhibit binding to red blood cells.

The discernable antigenic difference observed between egg- and mammalian cell-grown A/Switzerland/9715293/2013 reference viruses by one-way HI testing (Table 5) prompted us to select this NH 2015-16 vaccine strain for additional two-way antigenic characterization. Ferret antisera raised against two synthetic viruses expressing either the mammalian cell- or egg-derived antigen were tested with conventional reference strains and with synthetic viruses made on different backbones (Table 5). The two-way HI data confirmed that the synthetic viruses were antigenically similar to their corresponding reference strains, with HI titer differences within 2-fold. Ferret antisera raised against viruses with one backbone also effectively recognized viruses rescued on a different backbone. Interestingly, the cell-derived synthetic and wild-type antigens reacted better to antiserum raised to the synthetic virus expressing egg-adapted HA (RG-PS-2404) than to antiserum raised to the egg-adapted reference virus (≤2-fold versus 4 to 8-fold lower than the homologous titers, respectively). It is plausible that high growth backbones affect the amount of HA incorporation into virions, which could affect their avidity for red blood cells and/or the amount of antibodies elicited by ferret infection with such viruses. Both of these factors could impact the dynamics of the HI assay and are under investigation. Overall, we have demonstrated the ability of synthetic technology to generate viruses that are more antigenically similar to the mammalian cell-grown prototype compared to current egg-adapted vaccine viruses.

## 4 Discussion

Reports of antigenic differences between recommended CVVs and circulating viruses in recent years, particularly for H3N2 strains [4], [5], [6], [13], [14], has heightened public awareness and concern over the effectiveness of seasonal influenza vaccines. This concern highlights the need for an improved CVV generation system that is not reliant on legacy, egg-based technology. Antigenic differences may result from antigenic drift in circulating viruses, egg-adaptive mutations, or both. Between 2010 and 2014, HA mutations in the egg-adapted H3N2 vaccine strains resulted in antigenic changes that were associated with low vaccine effectiveness [4], [13]. In 2014–2015, the drifted A/Texas/50/2012 (H3N2) vaccine strain was antigenically distinct from dominant circulating strains [6], [14]. Furthermore, variability of H3N2 influenza virus isolation rates in eggs in recent years [22], [23] can add additional risk of the introduction of egg-adaptive changes that have an impact on the antigenicity of the vaccine virus within the current system. Therefore, the use of CVVs isolated or synthetically generated in certified mammalian cells could help increase the number of viruses available for CVV selection and, in some circumstances, provide viruses for vaccine manufacture that better correspond antigenically to the circulating strains.

MDCK cells have both α-2,6- and α-2,3-linked sialic acids on their surfaces, making them a more neutral substrate with respect to selection of altered variants of influenza virus [11]. Sequence analysis of influenza viruses in clinical samples and their laboratory-passaged derivatives have confirmed that MDCK cells are more likely than chicken eggs to maintain the prevalent HA genotypes present in clinical material [16]. MDCK cell-grown viruses are also more antigenically similar to the viruses replicating in humans than their egg-grown counterparts, as evidenced by their greater recognition by neutralizing and HI antibodies in post-infection human sera [24], [25].

To that end, we have established a synthetic system for generating CVVs exclusively in MDCK cells from sequence information [18]. This system has the potential to improve antigenic similarity to strains that cause human disease. For each new influenza A virus, a set of high-growth backbones can be tested empirically to identify the highest-yielding option for that particular HA and NA in mammalian cells and eggs. The backbones that we have established for influenza A viruses can increase virus rescue efficiency in MDCK cells and HA yields in both MDCK cells and in eggs [18]. This synthetic technology, combined with a standard PR8 backbone, has already been used to produce an H7N9 vaccine candidate that had a good safety profile and elicited antibody titers considered protective in a phase I trial [26]. Demonstrating that alternative backbones do not interfere with the antigenic properties of synthetic vaccine viruses would help facilitate the use of higher yielding chimeric backbones to produce CVVs for human vaccines.

In this study, we have shown that synthetic viruses could be produced that antigenically correspond to either egg- or mammalian cell-propagated isolates, based solely on the HA and NA sequences chosen for gene synthesis. Seasonal viruses generated with synthetic DNA and alternative backbones were passaged up to three times in a vaccine-approved MDCK cell line, and antigenic stability was established by HI titers comparable to those of the corresponding conventional reference strains. Although it is plausible that differences in the internal genes could affect certain physical properties of the virion, such as morphology or HA incorporation, which could in turn affect the dynamics of the HI assay, we have confirmed that the different backbones had no significant effect on antigenic characterization of the viruses. This finding was not unexpected since antigenicity should merely reflect the HA that has been introduced into the synthetic virus.

Although viruses passaged in MDCK cells generally do not acquire adaptive mutations in HA, some H3N2 viruses (which may have HA molecules with low affinity for cell surface sialic acid) have been reported to acquire upon passage a mutation in the NA sialic acid binding site that facilitates binding to MDCK cells [27]. Although the selected NA mutation does not interfere with the antigenic and immunogenic properties of the mutated viruses, per se, the altered properties of NA result in NA-mediated hemagglutination and consequent changes to HI titers. To block this NA-mediated hemagglutination, the NA inhibitor oseltamivir was added to the HI assays for all H3N2 strains in the studies we are reporting.

The ability of synthetic seed technology to increase the number of strains with new HAs and NAs that can be isolated and to provide strains that are more similar to a mammalian cell-grown prototype virus may improve antigenic similarity between the vaccine and viruses circulating in humans. In addition, the ability to generate CVVs rapidly using this technology compared to egg-based reassortment [18] could potentially allow strain recommendations to be made later, thus reducing the lag time between strain selection and vaccine distribution, during which antigenic drift may occur. The relative clinical effectiveness of vaccines that better corresponds to viruses in circulation still needs to be determined. Although antigenic characterization by HI provides important information about whether a vaccine made using the vaccine virus will protect against circulating strains, there are some limitations to using this methodology as a surrogate for immunogenicity or vaccine effectiveness. In particular, post-infection antisera raised against egg- and cell-derived vaccine viruses in immunologically naïve ferrets may not necessarily reflect the antibody repertoire elicited by inactivated and split vaccines in humans with pre-existing immunity. Nevertheless, the exclusive use of mammalian cells for generating CVVs and for antigen manufacture could reduce the potential risks associated with egg-adaptive mutations in influenza vaccine antigens.

## Figures and Tables

**Table 1:** HI comparison of egg-propagated non-synthetic viruses and MDCK cell-propagated synthetic viruses with HA and NA sequences derived from egg-adapted candidate vaccine viruses.

Origin^a^	Virus^b^	Genetic group	Passage history	Hemagglutination inhibition titer									
				Ferret antisera raised to egg-propagated viruses^c^									
				NIB-74	A/CC/16	IVR-165	A/Vic/361	X-187	A/Vic/210	X-175 C	A/Urug/716	IVR-164	A/Bris/299
				NIBSC F66/10	F30/10	NIB F11/12	F06/12	CDC 2010-098/10/101	F10/11	F1082/08	F26/08	NIB F10/12	NIBSC F12/12
CVV	NIB-74	A(H1N1)	Egg (E1/E3)	**20480**	20480								
WT	A/Christchurch/16/2010		Egg (E5/E1)	20480	**20480**								
S	RG-PS-1259 (NIB-74) PR8x		Cell (MDCK2)	20480	10240								
S	RG-PS-1260 (NIB-74) #19		Cell (MDCK2)	20480	20480								
S	RG-PS-1261 (NIB-74) #21		Cell (MDCK2)	20480	20480								

CVV	IVR-165	A(H3N2)	Egg (E3/D6/E1)			**1280**	2560						
WT	A/Victoria/361/2011	Clade 3C.1	Egg (E3/E2)			640	**1280**						
S	RG-PS-1291 (IVR-165) PR8x		Cell (MDCK3)			1280	1280						
S	RG-PS-1242 (IVR-165) #19		Cell (MDCK3)			2560	2560						
S	RG-PS-1252 (IVR-165) #21		Cell (MDCK3)			1280	2560						

CVV	X-187	A(H3N2)	Egg (Ex/E2/E1)					**5120**	2560				
WT	A/Victoria/210/2009		Egg (E2/E2)					5120	**2560**				
S	RG-PS-1274 (X-187) PR8x		Cell (MDCK2)					2560	1280				
S	RG-PS-1266 (X-187) #19		Cell (MDCK2)					5120	640				
S	RG-PS-1267 (X-187) #21		Cell (MDCK2)					2560	1280				

CVV	X-175C	A(H3N2)	Egg (Ex/E1/E1)							**2560**	1280		
WT	A/Uruguay/716/2007		Egg (SPCFK1/E5)							5120	**2560**		
S	RG-PS-2385 (X-175C) PR8x		Cell (MDCK1)							2560	640		
S	RG-ID-733 (X-175C) #19		Cell (MDCK2)							2560	1280		
S	RG-PS-2358 (X-175C) #20		Cell (MDCK1)							2560	1280		

CVV	IVR-164	A(H3N2)	Egg (E5/C8/E1)									**5120**	2560
WT	A/Brisbane/299/2011	Clade 6	Egg (E5/E2)									5120	**5120**
S	RG-PS-1472 (IVR-164) PR8x		Cell (MDCK1)									5120	5120
S	RG-PS-1468 (IVR-164) #19		Cell (MDCK1)									5120	5120
S	RG-PS-1469 (IVR-164) #21		Cell (MDCK1)									5120	5120

a S, synthetic virus; CVV, egg-adapted high growth reassortant strain; WT, egg-propagated wild-type isolate, ^b,c^ NIB-74, IVR-165, X-187, X-175 C, and IVR-164 are the egg-adapted high growth reassortant strains of A/Christchurch/16/2010 (abbreviated as A/CC/16), A/Victoria/361/2011 (abbreviated as A/Vic/361), A/Victoria/210/2009 (abbreviated as A/Vic/210), A/Uruguay/716/2007 (abbreviated as A/Urug/716), and A/Brisbane/299/2011 (abbreviated as A/Bris/299), respectively. Homologous titers are indicated in bold and underlined.

**Table 2:** HI characterization of synthetic viruses with HA and NA sequences derived from egg- or mammalian-propagated wild-type viruses.

Origin^a^	Viruses^b^	Genetic group	Passage history	Hemagglutination inhibition titer			
				Ferret antisera raised to wild-type isolates^c^			
				A/Texas/50/12 egg	A/Berlin/93/11 egg	A/South Australia/3/11 cell	A/Victoria/210/09 cell
				F42/13	F10/12	AUG F20/08	T/C St Judes F712/14
WT	A/Texas/50/2012-egg	A(H3N2)	Egg (E5/E2)	**2560**			
S	RG-PS-1958 (A/Texas/50/2012-egg) PR8x	clade 3C.1	Cell (MDCK1)	2560			
S	RG-PS-1959 (A/Texas/50/2012-egg) #19		Cell (MDCK1)	2560			
S	RG-PS-2334 (A/Texas/50/2012-egg) #21		Cell (MDCK1)	1280			

WT	A/Berlin/93/2011-egg	A(H3N2)	Egg (NVD3/E3)		**2560**	640	
S	RG-PS-1425 (A/Berlin/93/2011-egg) PR8x	clade 3C	Cell (MDCK1)		10240	2560	
S	RG-PS-1286 (A/Berlin/93/2011-egg) #19		Cell (MDCK1)		5120	640	
S	RG-PS-1287 (A/Berlin/93/2011-egg) #21		Cell (MDCK1)		10240	2560	

WT	A/South Australia/3/2011-cell	A(H3N2)	Cell MDCK2/SIAT2		640	**2560**	
S	RG-PS-1322 (A/South Australia/3/2011-cell) PR8x	clade 3C	Cell (MDCK1)		1280	5120	
S	RG-PS-1323 (A/South Australia/3/2011-cell) #19		Cell (MDCK1)		640	2560	
S	RG-PS-1327 (A/South Australia/3/2011-cell) #21		Cell (MDCK1)		2560	5120	

WT	A/Victoria/210/2009-cell	A(H3N2)	Cell (MDCK2/SIAT5)				**2560**
S	RG-PS-2378 (A/Victoria/210/2009-cell) PR8x		Cell (MDCK1)				2560
S	RG-KD-038 (A/Victoria/210/2009-cell) #19		Cell (MDCK1)				5120
S	RG-PS-2381 (A/Victoria/210/2009-cell) #21		Cell (MDCK1)				5120

a S, synthetic virus; WT, wild-type isolate. ^b,c^^“^Egg” or “cell” refers to the passage history of the wild-type viruses or the passage history of the reference viruses that provided the HA and NA sequences to make the synthetic viruses. In cases of mixed passage history, any passage in eggs is sufficient to trigger an “egg” designation. Homologous titers are indicated in bold and underlined.

**Table 3:** HI titers showing antigenic mismatch between viruses with egg- and mammalian cell-derived H3N2 antigens.

(A) A/Victoria/210/2009 (H3N2)					
	Antigen source^a^	Passage history	Post-infection ferret antisera		
			X-187 (A/Vic/210/09)-egg	A/Vic/210/09-egg	A/Vic/210/09-cell
			CDC 2010-098/10/101 pool	F10/11	T/C St Judes F712/14
Reference viruses					
X-187 (A/Victoria/210/2009)-egg	Egg	Egg (Ex/E2/E1)	**5120**	2560	2560
A/Victoria/210/2009-egg	Egg	Egg (E2/E2)	5120	**2560**	2560
A/Victoria/210/2009-cell	Cell	Cell (MDCK1/SIAT2)	640	320	**2560**

Synthetic test viruses					
RG-PS-1274 (X-187 A/Victoria/210/2009-egg) PR8x	Egg	Cell (MDCK2)	2560	1280	640
RG-PS-1266 (X-187 A/Victoria/210/2009-egg) #19	Egg	Cell (MDCK2)	5120	640	1280
RG-PS-1267 (X-187 A/Victoria/210/2009-egg) #21	Egg	Cell (MDCK2)	2560	1280	1280
RG-PS-2378 (A/Victoria/210/2009-cell) PR8x	Cell	Cell (MDCK1)	1280	640	2560
RG-KD-038 (A/Victoria/210/2009-cell) #19	Cell	Cell (MDCK1)	2560	640	5120
RG-PS-2381 (A/Victoria/210/2009-cell) #21	Cell	Cell (MDCK1)	2560	1280	5120

(B) A/Victoria/361/2011 (H3N2)					
	Antigen source^a^	Passage history	Post-infection ferret antisera		
			IVR-165 (A/Vic/361/11)-egg	A/Vic/361/11-egg	A/Vic/361/11-cell
			NIB F11/12	F06/12	F09/12
Reference viruses					
IVR-165 (A/Victoria/361/2011)-egg	Egg	Egg (E3/D6/E1)	**1280**	1280	2560
A/Victoria/361/2011-egg	Egg	Egg (E3/E2)	640	**2560**	5120
A/Victoria/361/2011-cell	Cell	Cell (MDCK2/SIAT5)	80	320	**320**

Synthetic test viruses					
RG-PS-1291 (IVR-165 A/Victoria/361/2011-egg) PR8x	Egg	Cell (MDCK3)	1280	1280	2560
RG-PS-1242 (IVR-165 A/Victoria/361/2011-egg) #19	Egg	Cell (MDCK3)	2560	2560	2560
RG-PS-1252 (IVR-165 A/Victoria/361/2011-egg) #21	Egg	Cell (MDCK3)	1280	2560	2560
RG-PS-1247 (A/Victoria/361/2011-cell) PR8x	Cell	Cell (MDCK3)	160	1280	1280

a “Egg” or “cell” refers to the passage history of the reference viruses or the passage history of the viruses that provided the HA and NA sequences to make the synthetic viruses. Homologous titers are indicated in bold and underlined.

**Table 4:** HI titers showing antigenic mismatch between viruses with egg- and mammalian cell-derived B antigens.

	Antigen source^a^	Passage history	Post-Infection ferret antisera	
			B/Brisbane/60/08-egg	B/Brisbane/60/08-cell
			F30/10	NIBSC66/10
Reference viruses				
B/Brisbane/60/2008-egg	Egg	Egg (E4/E3)	**640**	160
B/Brisbane/60/2008-cell	Cell	Cell (MDCKx/MDCK4)	20	**160**

Synthetic test viruses				
RG-ID-1279 (B/Brisbane/60/2008-egg)	Egg	Cell (MDCK1)	640	160
RG-PS-1376 (B/Brisbane/60/2008-cell)	Cell	Cell (MDCK3)	40	160

a “Egg” or “cell” refers to the passage history of the reference viruses or the passage history of the viruses that provided the HA and NA sequences to make the synthetic viruses. Homologous titers are indicated in bold and underlined.

**Table 5:** Two-way HI test for the H3N2 strain A/Switzerland/9715293/2013.

	Antigen source^a^	Passage history	Post-Infection ferret antisera^b^			
			A/Switz/9715293	A/Switz/9715293	A/Switz/9715293	A/Switz/9715293
			(WT) egg	(WT) cell	(S) egg RG-PS-2404	(S) cell RG-ID-1985
			NIBSC F29/15	NIBSC F13/14	F40/14	F439/14
Reference viruses						
A/Switzerland/9715293/2013-egg	Egg	E4/E1 clone 123	**640**	160	1280	160
A/Switzerland/9715293/2013-cell	Cell	SIAT1/SIAT3	80	**320**	320	320

Test viruses						
RG-PS-2404 (A/Switzerland/9715293/2013-egg) PR8x	Egg	MDCK1	320	160	**640**	160
RG-PS-2411 (A/Switzerland/9715293/2013-egg) #21	Egg	MDCK1	320	160	640	160
RG-PS-2407 (A/Switzerland/9715293/2013-cell) PR8x	Cell	MDCK1	160	320	320	320
RG-ID-1985 (A/Switzerland/9715293/2013-cell) #21	Cell	MDCK1	160	640	640	**640**
RG-PS-2400 (A/Switzerland/9715293/2013-cell) #19	Cell	MDCK1	160	320	320	320

a “Egg” or “cell” refers to the passage history of the reference viruses or the passage history of the viruses that provided the HA and NA sequences to make the synthetic viruses.

b Post-infection ferret antisera was raised to either wild-type isolates (WT) or synthetic viruses (S) Homologous titers are indicated in bold and underlined.
