# Supplementary material for: Antigenic characterization of influenza viruses produced using synthetic DNA and novel backbones
Source: Vaccine. 2016 Jul 12;34(32):3641–8. doi: 10.1016/j.vaccine.2016.05.031 (PMC4940205; doi:10.1016/j.vaccine.2016.05.031)
Supplement: Supplementary file 1 [file mmc1.docx]

**Table S1: Panel of synthetic viruses generated for antigenic characterization**

| **Virus strain** | **Antigen source^1^** | **Flu A Backbones** | | |
| --- | --- | --- | --- | --- |
|  |  | **PR8x** | **#19** | **#21** |
| A/Christchurch/16/2010 (H1N1) – NIB74 | egg | RG-PS-1259 | RG-PS-1260 | RG-PS-1261 |
| A/Brisbane/299/2011 (H3N2) - IVR164 | egg | RG-PS-1472 | RG-PS-1468 | RG-PS-1469 |
| A/Uruguay/716/2007 (H3N2) - X175C | egg | RG-PS-2385 | RG-ID-733 | RG-PS-2358 |
| A/Texas/50/2012 (H3N2) | egg | RG-ID-1958 | RG-ID-1959 | RG-PS-2334 |
| A/Berlin/93/2011 (H3N2) | egg | RG-PS- 1425 | RG-PS-1286 | RG-PS-1287 |
| A/South Australia/3/2011 (H3N2) | cell | RG-PS-1322 | RG-PS-1323 | RG-PS-1327 |
| A/Victoria/210/2009 (H3N2) - X187 | egg | RG-PS-1274 | RG-PS-1266 | RG-PS-1267 |
| A/Victoria/210/2009 (H3N2) | cell | RG-PS-2378 | RG-KD-038 | RG-PS-2381 |
| A/Victoria/361/2011 (H3N2) - IVR165 | egg | RG-PS-1291 | RG-PS-1242 | RG-PS-1252 |
| A/Victoria/361/2011 (H3N2) | cell | RG-PS-1247 | n/a | n/a |
| A/Switzerland/9715293/2013 (H3N2) | egg | RG-PS-2404 | n/a | RG-PS-2411 |
| A/Switzerland/9715293/2013 (H3N2) | cell | RG-PS-2407 | RG-PS-2400 | RG-ID-1985 |
|  |  | **Flu B Backbone** | | |
| B/Brisbane/60/2008 | egg | RG-ID-1279 | | |
| B/Brisbane/60/2008 | cell | RG-PS-1376 | | |

^1^ “egg” or “cell” under antigen source refers to the passage history of the viruses that provided the HA and NA sequences for synthesis. In cases of mixed passage history, any passage in eggs is sufficient to trigger an “egg” designation. All synthetic test viruses were passaged exclusively in mammalian cells for these studies, regardless of the HA and NA sequences used.

**Table S2: Accession Numbers of HA and NA sequences used to generate synthetic viruses**

| **Virus strain** | **Antigen source** | **HA sequence** | **NA sequence** | **Originating lab^1^** |  |
| --- | --- | --- | --- | --- | --- |
| A/Christchurch/16/2010 (H1N1) - NIB74 | egg | EPI280344 | EPI280343 | WHO CC |  |
| A/Brisbane/299/2011 (H3N2) - IVR164 | egg | EPI358048 | EPI358049 | WHO CC |  |
| A/Uruguay/716/2007 (H3N2) - X175C | egg | EPI162118 | EPI171971 | NYMC |  |
| A/Texas/50/2012 (H3N2) | egg | EPI408130 | EPI408131 | CDC |  |
| A/Berlin/93/2011 (H3N2) | egg | EPI359596^2^ | EPI359595 | NIMR |  |
| A/South Australia/3/2011 (H3N2) | cell | EPI333046 | EPI333045 | IMVS |  |
| A/Victoria/210/2009 (H3N2) - X187 | egg | EPI244232 | EPI244231 | NYMC |  |
| A/Victoria/210/2009 (H3N2) | cell | EPI269899^3^ | EPI269898 | VIDRL |  |
| A/Victoria/361/2011 (H3N2) - IVR165 | egg | EPI551807 | EPI551805 | CSL |  |
| A/Victoria/361/2011 (H3N2) | cell | EPI377442 | EPI551805 | WHO CC |  |
| A/Switzerland/9715293/2013 (H3N2) | egg | EPI537866 | EPI537865 | NIMR |  |
| A/Switzerland/9715293/2013 (H3N2) | cell | EPI530687 | EPI530688 | HCUG |  |
| B/Brisbane/60/2008 | egg | KX058884^4^ | EPI173276 | QHSS |  |
| B/Brisbane/60/2008 | cell | EPI163725 | EPI173276 | QHSS |  |

^1^Influenza HA and NA sequences were obtained from GISAID, unless otherwise indicated; originating labs are indicated below:

CDC - Centers for Disease Control and Prevention, Atlanta, GA

CSL – CSL, Australia

HCUG – Hopital Cantonal Universitaire de Geneves, Switzerland

IMVS – Institute of Medical and Veterinary Science, Australia

NIMR – National Institute for Medical Research, UK (now The Francis Crick Institute, Mill Hill Laboratory, UK)

NYMC – New York Medical College, NY

QHSS – Queensland Health Scientific Services, Australia

VIDRL – Victorian Infectious Diseases Reference Laboratory, Australia

WHO CC - WHO Collaborating Centre for Reference and Research on Influenza, Melbourne, Australia

^2^EPI359596 had an ambiguous purine at nucleotide position 604. A guanosine was selected for synthesis based on sequencing of the actual virus.

^3^EPI269899 had a thymidine at nucleotide position 616. The recommendation based on personal communication was to change to a cytidine for gene synthesis.

^4^This HA sequence was not obtained from GISAID. GenBank accession number provided.

**Table S3: Amino acid differences in synthetic egg- and mammalian cell-derived paired antigens**

|  | **position** | **cell** | **egg** |
| --- | --- | --- | --- |
| A/Victoria/210/2009 (H3N2) | 186 | G | V |
|  | 228 | S | T |
| A/Victoria/361/2011 (H3N2) | 156 | H | Q |
|  | 186 | G | V |
|  | 219 | S | Y |
| A/Switzerland/9715293/2013 (H3N2) | 140 | I | R |
|  | 186 | G | V |
| B/Brisbane/60/2008 | 199 | T | A |

1. **B.**

**C. D.**

**Figure S1: Virus growth comparison of synthetic viruses made with egg- and mammalian cell-derived paired antigens.** Influenza viruses containing egg- or mammalian cell-derived synthetic HAs and NAs were used to infect 7 mls of MDCK 33016PF cell culture for 72 hrs. Virus titers were determined with a focus formation assay for **(A)** A/Switzerland/9715293/2013 (H3N2), **(B)** A/Victoria/210/2009 (H3N2), **(C)** A/Victoria/361/2011 (H3N2), and **(D)** B/Brisbane/60/2008. Error bars indicate the standard error of the mean from at least 2 independent experiments wherein all measurements were made in duplicate.
